# A comparative evaluation of Advanced Platelet-Rich Fibrin (A-PRF) and Platelet-Rich Fibrin (PRF) as a Scaffold in Regenerative Endodontic Treatment of Traumatized Immature Non-vital permanent anterior teeth: A Prospective clinical study

**DOI:** 10.4317/jced.57902

**Published:** 2021-05-01

**Authors:** Veena Jayadevan, Paras-Mull Gehlot, Vinutha Manjunath, Subbarao V. Madhunapantula, Jyothi-Swandenahalli Lakshmikanth

**Affiliations:** 1 BDS. Department of Conservative Dentistry and Endodontics. JSS Dental College and Hospital. JSS Academy of Higher Education and Research. Sri Shivarathreeshwara Nagar. Mysuru- 570015, Karnataka, India; 2 MDS. Department of Conservative Dentistry and Endodontics. JSS Dental College and Hospital. JSS Academy of Higher Education and Research. Sri Shivarathreeshwara Nagar. Mysuru- 570015, Karnataka, India; 3 BSc, B.Ed, M.Sc, Ph.D. Department of Biochemistry. JSS Medical College and Hospital. JSS Academy of Higher Education and Research. Sri Shivarathreeshwara Nagar. Mysuru- 570015, Karnataka, India; 4 M Pharm. Department of Pharmaceutics. JSS College of Pharmacy. JSS Academy of Higher Education and Research. Sri Shivarathreeshwara Nagar. Mysuru- 570015, Karnataka, India

## Abstract

**Background:**

Regenerative endodontic treatment (RET) is a promising treatment alternative for traumatized immature non-vital teeth. Advanced platelet-rich fibrin (A-PRF) contains significantly more growth factors than Platelet-rich fibrin (PRF) and has not been evaluated as a scaffold in RET. The aim of the present study was to evaluate and compare A-PRF and PRF as scaffolds in the RET concerning periapical healing, and root development of traumatized immature non-vital teeth.

**Material and Methods:**

In the present study, RET was performed on 30 traumatized immature non-vital maxillary incisors in 28 patients aged between 8-27 years. Minimal mechanical debridement and irrigation with 1.5% sodium hypochlorite and 17% ethylenediaminetetraacetic acid was performed. Canals were disinfected using modified triple antibiotic paste consisting of ciprofloxacin, metronidazole and cefaclor. Based on the type of scaffold, teeth were randomly assigned into A-PRF (n=15) and PRF groups (n=15). Periapical healing, apical response and quantitative root dimensions (length and thickness) were analyzed radiographically after 12 months follow-up.

**Results:**

Nineteen patients with 21 teeth (A-PRF n=11, PRF n=10) completed the follow-up and 9 patients were excluded. Clinically, patients in both the groups were asymptomatic. The survival rates for A-PRF and PRF were 78.5% and 77.5%, respectively. No statistically significant differences were detected between A-PRF and PRF regarding periapical healing and type of apical response (*p*& 0.05). The difference in the pre-operative and follow-up root thickness and root length in both A-PRF and PRF groups were statistically significant (*p*< 0.05).

**Conclusions:**

Based on short-term results of 13 months, both A-PRF and PRF can be used as scaffold in regenerative endodontic treatment of traumatized immature non-vital teeth. A-PRF could be recommended in such cases since it yielded more root dentin thickness which is crucial for reinforcing immature teeth.

** Key words:**Regenerative endodontic treatment, dental trauma, Non-vital teeth, immature teeth, platelet-rich fibrin, advanced platelet-rich fibrin.

## Introduction

The management of an immature non-vital tooth is a challenge in Endodontics due to difficulty in performing chemo-mechanical debridement and creating an effective apical seal by conventional endodontic treatment (1).

Pulpal necrosis frequently occurs due to dental trauma in children (2). Apexification has been suggested in the management of immature non-vital tooth, which can induce apical closure through the formation of mineralized tissue in the apical region. However, apexification does not increase the root dentin thickness nor increase the root length/apical closure which eventually could lead to the fracture of the tooth (3,4).

Recently, Regenerative endodontic therapy (RET) has been recommended in management of immature tooth with necrotic pulp and/or apical periodontitis/abscess (5). RET is a “biologically-based procedures designed to physiologically replace damaged tooth structures, including dentin and root structures, as well as cells of the pulp-dentin complex” (6). The key components of RET include: (i) Stem cells mainly from Apical papilla (SCAPs), (ii) Growth factors/morphogens and (iii) Scaffold that can support cell growth and differentiation (5).

The ideal characteristics of a scaffold for successful regeneration have been summarized by Nosrat *et al* (7). The use of blood clot, platelet-rich plasma (PRP), and platelet-rich fibrin (PRF) as bioscaffolds has been reported in literature (8,9). PRP is a natural scaffold that eliminates the risk of cross-infection and immunogenicity. However, it is not 100% autologous (10). PRF was introduced in RET to overcome this limitation of PRP and furthermore PRF also exhibited enhanced release of growth factors and leukocytes. With a better understanding of the use of autologous platelet concentrates and their role in healing, newer variants of PRF have been developed. Studies demonstrated that a slight increase in centrifugation time and reduction in speed resulted in the development of advanced platelet-rich fibrin (A-PRF), a variant of standard PRF with better regenerative potential, which is commonly used in periodontal regeneration and implant surgery (10,11).

A-PRF membrane have shown promising response in the endodontic surgery (12). However, the use of A-PRF as a scaffold in regenerative endodontics has not been attempted. Thus, this prospective clinical study aimed to evaluate and compare the treatment outcomes of A-PRF and PRF as a scaffold in RET of traumatized immature non-vital teeth.

## Material and Methods

This prospective clinical study was conducted according to the declaration of Helsinki (1975) regarding biomedical research involving human subjects and the protocol was approved by the institutional ethics committee.

The study was performed from April 2018 to September 2019 at a single Centre of Department of Conservative Dentistry and Endodontics. The study design was randomized with a two-arm, parallel design (1:1 allocation ratio). Regenerative endodontic treatments (RET) were performed either using A-PRF or PRF as a scaffold.

Patients reporting to the outpatient of the Department of Conservative Dentistry and Endodontics and Department of Pediatric Dentistry were enrolled. Patients aged between 8 and 30 years meeting the inclusion criteria were randomly allotted to the two groups of A-PRF (n=15) and PRF (n=15). Cooperative patients with an immature single-rooted non-vital tooth with apical width more than 1 mm, with or without periapical lesion, and trauma as an etiology, with no systemic diseases were included in the study. Teeth with attempted access cavity without intracanal medicament placement or two teeth per participant could be included in the study. Teeth with mature apex, internal or external resorption, mobility, ankylosis, root fracture, periodontal pocket, and unrestorable structure were excluded from the study. Furthermore, patients with known allergy to antibiotics and local anesthetic agents were also excluded from the study.

Participants were informed about the purpose, procedure, and expected outcome of the study along with alternate treatment options. A signed informed assent/consent form was obtained from each patient or parent.

-Clinical procedures

A preoperative diagnosis of pulp necrosis was established based on the electric pulp test (Parkell, Inc., NY, USA) and cold testing with Roeko Endo-Frost (Coltene/Whaledent GmbH, Germany). A two-visit RET was performed by a single operator. The clinical protocol for RET procedure was in accordance with the American Association of Endodontists (AAE) guidelines (13,14).

First visit:

Local anesthesia containing 2% lignocaine hydrochloride and 1:80,000 adrenaline (Lignox A, Indoco Remedies Ltd., Gujarat, India) was administered and access cavity prepared under rubber dam isolation. Pulpal remnants were extirpated using barbed broaches and copious, gentle irrigation with 1.5% sodium hypochlorite (NaOCl) (Nice Chemicals (P) Ltd., Kochi, India) (20 mL/canal, 5 min) using a 30-G side venting needle (RC Twents, Prime Dental. Pvt Ltd., Mumbai, India) about 2 mm short of the root end. The canal was then irrigated with saline (Denis Chem Lab Limited, Kalol, India) and 17% ethylenediaminetetraacetic acid (EDTA) (Canalarge, Ammdent, India) (20 mL/canal, 5 min). Radiographic working length was determined using paralleling technique (Gendex Visualix, Dentsply, Italy). Minimal canal debridement was done using K-files (#80–120 size, Mani Inc., Japan). Canal was dried using paper points (DiaDent, Korea), and modified triple antibiotic paste was placed in the canal till the working length and coronally short of the cementoenamel junction (CEJ) using a lentulospiral. For preparation of the modified triple antibiotic paste (TAP), an equal proportion (1:1:1) of sugar-free Tablets of ciprofloxacin, metronidazole (Albert David Limited, WB, India), and cefaclor (Health biotech limited, Chandigarh, India) was powdered and mixed with 1 ml of propylene glycol (vehicle) to obtain a stock solution of 100mg/ml, which was diluted to obtain a final concentration of 1mg/mL (8,15). The access cavity was sealed with a temporary restorative material of at least 3–4 mm thickness (Cavit. 3M ESPE, St. Paul, MN, USA). Patients were recalled after 4 weeks.

Second Visit (After 4 weeks):

The patients were assessed for any signs or symptoms of persistent infection, and the intracanal medicament was repeated if required. Asymptomatic patients were recruited for RET.

Plain local anesthesia with 3% Mepivacaine (Scandonest, Septodont, Canada) without vasoconstrictor was administered. All the steps were performed using dental operating microscope (OPMI Pico, Carl Zeiss, Oberkochen,Germany). The tooth was re-accessed under rubber dam isolation. Copious and gentle irrigation with saline and 20 ml of 17% EDTA was performed to remove residue of the TAP, and paper points were used to dry the canal. Bleeding was induced in the canal system by overinstrumentation and rotation of a slightly precurved K-file (#40 size, Mani Inc., Japan) at 2 mm past the apical foramen with the aim of having the canal filled with blood to the level of the CEJ. A standard venipuncture was performed (median cubital vein). A-PRF or PRF was freshly prepared using a centrifuge (R-8C Laboratory centrifuge, Remi Lab, Mumbai, India). For PRF, 10 ml of intravenous blood was drawn into a tube without anticoagulant and centrifuged at 2700 rpm for 12 min (10). For A-PRF, 10 ml of intravenous blood was drawn into a tube without anticoagulant and centrifuged at 1500 rpm for 14 min (11).

The fibrin clots from both A-PRF and PRF were removed from the tubes and separated from the red element phase below the buffy coat using sterile scissors. The clot was delicately placed inside a sterile box (Magic PRF & GRF Box, Jull-Dent, Mumbai, India) and squeezed between sterile metal plates to obtain a membrane. The A-PRF or PRF clot was then placed into the root canal using an endodontic plugger. Biodentine (Septodont, Saint-Maur-des-Fossés, France) was placed as a capping material over the A-PRF or PRF. Glass ionomer cement (GIC) (GC Fuji IX, GC India) was placed gently in a thickness of about 3–4 mm over the Biodentine and the access was temporized with Cavit. A postoperative radiograph was taken to evaluate the placement of Biodentine and orifice barrier.

Patients were dismissed with instructions about the follow-up and for reporting in case of any symptoms. In cases where intracanal bleeding was not achieved, patients were placed into the rescue group to undergo conventional Biodentine apexification. Post regenerative treatment consisted of non-vital bleaching or composite restoration. These procedures were performed after a period of one week.

-Clinical and Radiographic evaluations 

Patients were reviewed after 6 months (±2 weeks) and 12 months (±2 weeks) clinically, and radiographs were taken using the same x-ray device and paralleling technique by the same operator. During the 12-month follow-up period, 9 patients were excluded from the study. Three radiographic evaluations were done at the end of the 12 months of follow-up.

(i) Periapical status

The periapical healing was evaluated using the periapical index (PAI) given by Orstavik *et al.* (16). The PAI scores of the preoperative and postoperative radiographic images were assigned by a single investigator for all the cases.

Treatment outcomes were categorized as follows (17): (a) Healed: Both the clinical (subjective and objective) and radiographic presentations (PAI score 1 or 2) were normal (b) Healing: The periapical radiolucency was reduced (PAI score 3 or 4) with a normal clinical presentation and (c) Diseased: The radiolucency had either increased or persisted without change (PAI score increased or unchanged) even when the clinical presentation was normal, or the clinical signs or symptoms were present regardless of the radiographic presentation.

(ii) Determination of changes in the root length and dentin thickness.

The preoperative and postoperative root length and dentin thickness were measured using the open source software, ImageJ (National Institutes of Health, Bethesda, MD). The TurboReg plugin (Biomedical Imaging Group, Swiss Federal Institute of Technology, Lausanne, VD, Switzerland) was used with the software to minimize any dimensional change due to angulation errors in the preoperative and postoperative radiographs (18). The image sizes were calibrated to the size #2 of an intraoral radiographic film, which facilitated the measurement of changes in root size on a millimeter (mm) scale (18,19).

**Figure 1:**
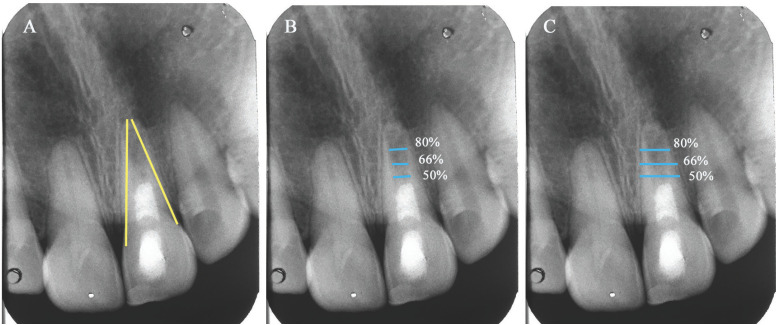
Radiographic root measurement methods, using ImageJ software. (A) Root length measurement (B) pulp space width measurement, and (C) total root width measurement. The root thickness was determined subtracting pulp space width from the total root width.

The root length and thickness were measured using the ‘‘straight-line’’ tool of TurboReg. A modified protocol proposed by Alobaid *et al*. was applied for radiographic measurements (20). Accordingly, the measurements for root length were performed as a straight line from the CEJ to the midpoint of the apex of the root from both the mesial and distal points, and then both measurements were averaged to obtain the total root length (Fig. 1A). The root width was measured at three levels: 50%, 66%, and 80% of root length. The dentin thickness was determined by subtracting the average pulp space width (mm) from the average total root width (mm) (Fig. 1B,C).

**Figure 2:**
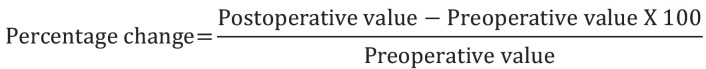
Formula.

The above protocol was used to determine the preoperative and postoperative radiographic measurements. The percentage change in the radiographic dimensions of preoperative and postoperative stages was calculated using the formula (21), (Fig. 2):

Two calibrated investigators viewed and performed the measurements. Inter-examiner reliability was measured using Pearson’s correlation (r = 0.948), indicating the reliability of the measures. The average of both the measurements obtained by the two investigators was considered as the final value for each radiographic outcome.

(iii) Apical response of immature tooth.

The apical response of the immature tooth to RET was evaluated according to Chen *et al.* (5). Type 1: Increased thickening of the canal walls and continued root maturation. Type 2: No significant continuation of root development with the root apex becoming blunt and closed. Type 3: Continued root development with the apical foramen remaining open. Type 4: Severe calcification (obliteration) of the canal space. Type 5: Hard tissue barrier formed in the canal space between the coronal Biodentine plug and the root apex.

-Statistical Analysis 

The data was tabulated and analyzed by parametric tests (t-test and chi-square test) to determine the differences in the root length and root dentin thickness for A-PRF and PRF group. Statistical significance was set at *p*<0.05. The data analysis was done using the Statistical Package for Social Sciences, version 21 (SPSS Inc., Chicago, IL).

## Results

**Figure 3:**
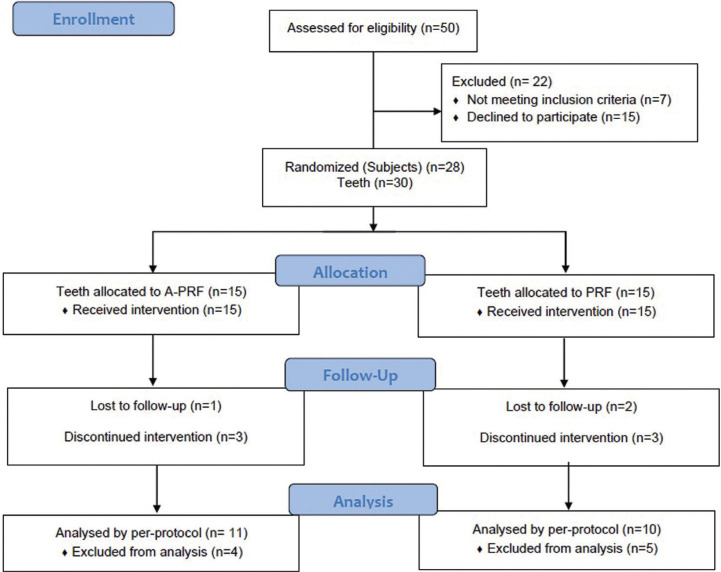
Study flow diagram.

Table 1 summarizes the characteristics of the study population. The participant’s flow diagram of the study is presented in Fig. 3. A total of 19 patients with 21 teeth (A-PRF n=11, PRF n=10) completed the 12 months follow-up. The exclusions in A-PRF were because of secondary fracture in one patient, pain in two patients, and one patient lost to follow-up. Hence, the survival rate for teeth treated with A-PRF was 78.5% (11/14). The exclusions in PRF group were because of pain in three cases and two patients lost to follow-up. The reason for the patients lost to follow-up was not known. Thus, the survival rate for teeth in the PRF group was 77% (10/13). The clinical and radiographic parameters before and after the RET are summarized in Table 2.

**Table 1:**
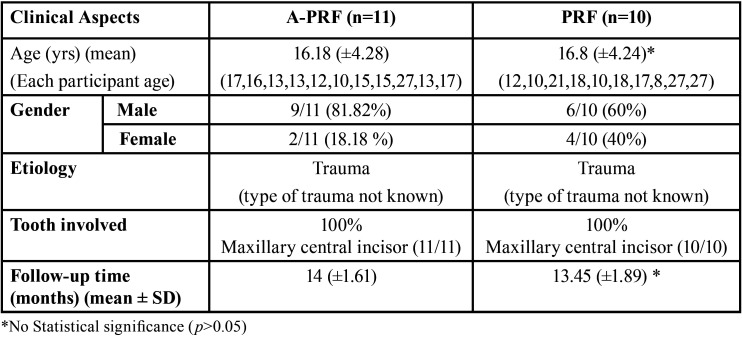
A Summary of patient demographics.

**Table 2:**
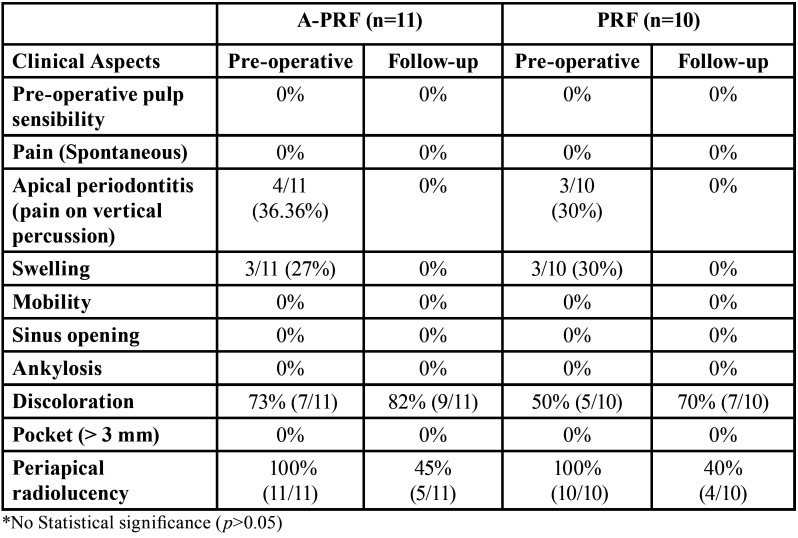
Clinical and Radiographic parameters before and after RET in A-PRF and PRF group.

**Figure 4:**
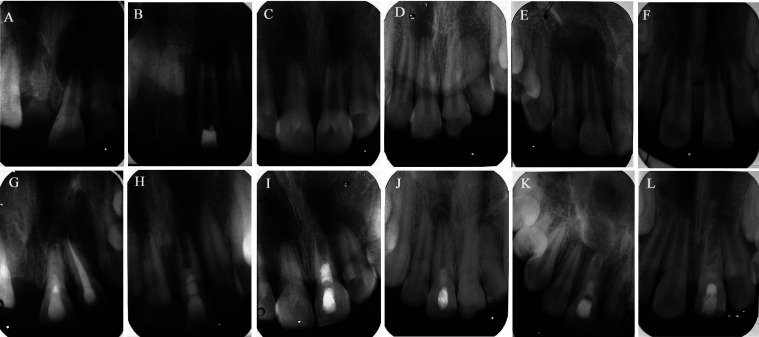
Pre-operative radiographs with periapical lesion and immature apex (A,B and C for A-PRF and D,E and F for PRF) and final follow-up respective radiographs (G,H and I for A-PRF and J, K and L for PRF). (G) #21, A-PRF treated, 20-year-old male with a 16-month follow-up shows periapical healing with increased root thickening and root maturation. (H) #21, A-PRF treated, 17-year-old male with 17-months follow-up shows periapical healing with no significant continuation of root development. (I) #21, A-PRF treated, 12-year-old male, a 15-months follow-up reveals periapical healing with closed root apex (J) #11, PRF treated, 12-years-old male with 12 months follow-up, minimal periapical healing with thickening of canal walls and continued root maturation (K) #11, PRF treated, 17-year-old, male with 12-months follow-up, periapical healing and root apex closed (L) #21, PRF-treated, 8-year-old female, 12 months follow-up, periapical healing with root apex closed.

Considerable periapical healing was noted in the follow-up radiographs of both groups (Fig. 4); however, the difference in this parameter between the two groups was not statistically significant (*p* >0.05) (Table 3). No statistically significant difference was noted in the radiographic measurements between the two groups (*p* > 0.05) (Table 4). However, the A-PRF group showed greater root thickness on follow-up, and the PRF group showed greater root length, and these differences were statistically significant (*p* < 0.05) (Table 4).

**Table 3:**
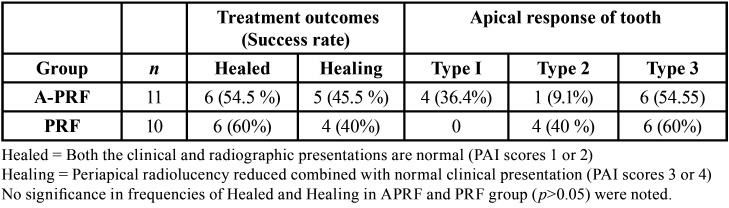
Treatment outcomes (Success) and apical response of immature tooth after treatment.

**Table 4:**
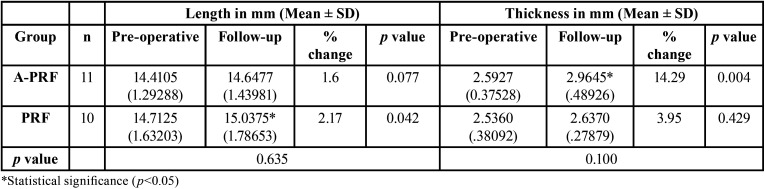
Mean radiographic root length, thickness and percentage change for pre-operative and follow-up images for A-PRF and PRF group.

Differences in the type of apical response were not significant among the groups (*p* > 0.05); however, the A-PRF group had more type I response compared to none in PRF (Table 3).

## Discussion

A scaffold forms a three-dimensional tissue structure and also play a key role in regulating stem cell differentiation by release of growth factors locally or by the signalling cascade triggered when stem cells bind to the extracellular matrix and to each other in a three-dimensional environment (2). In the present study, bleeding was induced from the periapical area, to promote the migration of the mesenchymal stem cell population into the root canal space (7,13,22).

Blood clot has been advocated and used as a scaffold in RET (19,23). However, few studies have found that in clinical practice, it was not always possible to induce enough blood to serve as a scaffold, which would eventually increase the possibility of sealing material collapse (24). Moreover, several studies have found the role of platelet concentrates to be advantageous (accelerated growth) in regeneration compared to only blood clot as scaffold (9). This is attributed to increased availability of growth factors in platelet concentrates which offer a longer and richer exposure leading to a better scaffold for cell differentiation and growth (25).

Kobayashi *et al.* examined the release of various growth factors from PRP, PRF and A-PRF at various intervals such as 15 min, 59 min, 8 h, 1 day, 3 days, and 10 days; and found that A-PRF released the maximum amount of growth factors over a longer duration when compared to either PRF or PRP which would be beneficial for regenerative procedures (26). Studies have demonstrated that A-PRF could function not only as a scaffold but also a reservoir capable of releasing certain growth factors at the site of application (27). TGF-β1 helps in the regulation of cell proliferation, differentiation, and reparative dentinogenesis. Wang *et al.* have shown that loss of TGF-β signaling in odontoblasts and bone producing mesenchyme results in failure of root elongation, reduced radicular dentin matrix density, and delayed molar eruption (28). A study reported a combination of SCAP, Bone morphogenetic protein 2 (BMP 2), and VEGF leading to the increased expression of osteo-/odontogenic differentiation-associated genes and protein and more mineralization deposits (29).

The success of RET has been reported in terms of periapical healing, increase in root thickness and/length, apical foramen closure and tooth survival (30). In the present study complete resolution (healed) of periapical lesion was noted in 54.5% (6/11) for A-PRF and 60% (6/10) for PRF group; and “healing” was noted in 45.5% (5/11) for A-PRF and 40% (4/10) for PRF as scaffold. At the end of 12 months follow-up all the patients were asymptomatic without signs and symptoms of active infection. Hence, A-PRF or PRF as a scaffold along with blood clot, induced a favorable environment for the periapical resolution.

Revascularization studies (inducing bleeding in canal) in immature teeth have reported 90-100% healing of periapical lesions with follow-up period ranging from 9-22 months (9,14,22). An *in vivo* animal study reported 100% reduction rates in the periapical lesion sizes with blood clot only and blood clot + PRF (23). A clinical study compared the performance of PRP, PRF, platelet pellet and blood clot as a scaffold, and found similar healing in all, over a 28 months follow-up (25).

Depending on the type of scaffold used, studies suggest a success of 80-94% in terms of root length and thickness (31). In the present study, for A-PRF group the increase in root lengths occurred in 72% (8/11) and root thickness in 91% (10/11); and for PRF group the root length increase occurred in 80% (8/10) and the thickness improved in 50% (5/10) of cases. Similarly, various revascularization studies on traumatized immature teeth reported 0-34.8% increase in root length and 43.5-45% increase in root thickness at a 12-19 months follow-up period (14,32). In contrast, Narang *et al.* reported a success rate of 99% for root length and 60% for dentinal wall thickness using PRF at 18 months follow-up (9).

Retrospective studies have reported a greater percentage change for root thickness (10-28.2%) compared to root length (14.9%) with a follow-up range of 10-21 months (19,20). In the present study the percentage change for root thickness and root length for A-PRF was 14.29% and 1.6% respectively and the results couldn’t be compared due to lack of similar studies. For PRF, the percentage change for root thickness and root length was 3.95% and 2.17% respectively which was less to compared to 11.14% (thickness) and 7.05% (length) reported at 28 months follow-up by a recent study (25). These differences could be attributed to follow-up period, etiology of non-vitality and the methods used for radiographic measurement (19,25,32). Further it is possible that with longer follow-up period the root dimensions would continue to increase (14,30). Although in the present study, the follow-up period was longer for A-PRF than PRF group, but this was not statistically significant. The sensibility test was negative in all the subjects which could be due to the coronal seal with Biodentine and GIC (30).

The difference in the outcome of A-PRF and PRF group could be due to the difference in age of the participants, since in A-PRF group only one subject was above 20 years, compared to PRF group with 3 subjects above 20 years. However, comparing the mean age of participants in both the groups, there was no statistical significance. Estefan *et al.* found that age had an influence on the increase in root length and root thickness (33). The role of age could be attributed to alteration of fibrin network patterns in younger patients and its interaction with platelets thus, influencing the quality of fibrin clot (34).

To eliminate operator bias, all the treatment steps were carried by a single operator. Further, paralleling device was used for radiographic technique and Alobaid method was used for all radiographic measurements. This method has the highest intra- and interobserver reliability (21). ImageJ software with TurboReg plug-in is sufficiently sensitive in estimating root length and thickness for regenerative procedures (18).

NaOCl (1.5%) was used as irrigant in the present study due to its minimal cytotoxicity to SCAP (13,31) and ability to release higher TGF-β1 levels from the canal walls when used along with 17% EDTA (35). Apart from removing the smear layer, the use of 17% EDTA promotes the expression of the odontoblast-like cell marker dentin sialophosphoprotein (DSPP) by 2.2-fold, increases the surface wettability of dentin thereby promoting the adherence of dental pulp stem cells on EDTA-treated dentin (36,37). Immature teeth have thin dentinal walls, hence minimal mechanical instrumentation was done for removal of biofilm (37). In the present study TAP at a concentration of 1mg/ml was used for canal disinfection, as it is highly effective against common bacterial flora of infected root canal space and is found to be safe for the stem cell survival (4). To reduce the risk of tooth discoloration minocycline was substituted by cefaclor (8). Although calcium hydroxide is recommended as an intracanal medicament in RET (4), a study by song *et al.* found more intracanal calcification in cases medicated with Calcium hydroxide (76.9%) compared with antibiotic paste (46.2%) (38).

Studies reveal the most likely outcome of RET in immature tooth is the increase in root thickness compared to length, and this has been attributed to the injury of Hertwig’s epithelial root sheath (HERS) cells which are responsible for increase in root length (5,30). Histologic characteristics of the root elongation and thickening were cementum-like tissue deposition at the apical region and lateral wall of the canal; and scattered bone-like tissue in the canal (23). Some complications which can occur following revascularization in immature permanent non-vital teeth are pulp canal calcification or ankylosis between the intracanal hard tissue and the apical bone (5). In the present study, 1 patient treated with A-PRF, presented with radiographic evidence of intracanal calcified layer between the Biodentine and the apex.

Biodentine was used as a capping material over the A-PRF/PRF scaffold, since it has a capacity to stimulate TGF-β1 release from the radicular dentin (39), shorter setting time compared to Mineral trioxide aggregate and also less discoloration (8).

The limitations of present study include: (i) small sample size (ii) the type of trauma in the participants could not be determined (iii) A-PRF and PRF were used concurrently with blood clot (iv) the radiographic evaluations were two-dimensional in nature (v) apical foramen closure was not included as a study parameter (vi) age and gender of subjects were not standardized and not uniformly distributed in groups and (vii) short follow-up period.

The results of the present study should be interpreted with caution since the teeth under investigation could not be studied histologically. Future *in vivo* studies and randomized controlled clinical trials with large sample size and longer follow-up are required to establish the role of A-PRF in the success of RET.

## Conclusions

The results of the present study showed that RET outcomes for traumatized immature non-vital teeth with A-PRF and PRF as scaffolds presented with similar clinical and radiographic findings at the end of 12 months of follow-up. A-PRF showed better root thickness than root length and PRF showed better root length than root thickness. Since gain in root thickness is crucial in immature teeth, A-PRF as a scaffold could be considered over PRF.
